# All-Atom Structural Models of the Transmembrane Domains of Insulin and Type 1 Insulin-Like Growth Factor Receptors

**DOI:** 10.3389/fendo.2016.00068

**Published:** 2016-06-20

**Authors:** Hossein Mohammadiarani, Harish Vashisth

**Affiliations:** ^1^Department of Chemical Engineering, University of New Hampshire, Durham, NH, USA

**Keywords:** molecular dynamics simulations, transmembrane domains, insulin receptor, insulin-like growth factor receptor, cell signaling, diabetes

## Abstract

The receptor tyrosine kinase superfamily comprises many cell-surface receptors including the insulin receptor (IR) and type 1 insulin-like growth factor receptor (IGF1R) that are constitutively homodimeric transmembrane glycoproteins. Therefore, these receptors require ligand-triggered domain rearrangements rather than receptor dimerization for activation. Specifically, binding of peptide ligands to receptor ectodomains transduces signals across the transmembrane domains for trans-autophosphorylation in cytoplasmic kinase domains. The molecular details of these processes are poorly understood in part due to the absence of structures of full-length receptors. Using MD simulations and enhanced conformational sampling algorithms, we present all-atom structural models of peptides containing 51 residues from the transmembrane and juxtamembrane regions of IR and IGF1R. In our models, the transmembrane regions of both receptors adopt helical conformations with kinks at Pro961 (IR) and Pro941 (IGF1R), but the C-terminal residues corresponding to the juxtamembrane region of each receptor adopt unfolded and flexible conformations in IR as opposed to a helix in IGF1R. We also observe that the N-terminal residues in IR form a kinked-helix sitting at the membrane–solvent interface, while homologous residues in IGF1R are unfolded and flexible. These conformational differences result in a larger tilt-angle of the membrane-embedded helix in IGF1R in comparison to IR to compensate for interactions with water molecules at the membrane–solvent interfaces. Our metastable/stable states for the transmembrane domain of IR, observed in a lipid bilayer, are consistent with a known NMR structure of this domain determined in detergent micelles, and similar states in IGF1R are consistent with a previously reported model of the dimerized transmembrane domains of IGF1R. Our all-atom structural models suggest potentially unique structural organization of kinase domains in each receptor.

## 1 Introduction

Insulin receptor (IR) and type 1 insulin-like growth factor receptor (IGF1R) are homologous, ligand-activated, and constitutively homo-dimeric transmembrane glycoproteins of the receptor tyrosine kinase (RTK) superfamily (1). Both IR and IGF1R have similarities in primary sequences, structural topologies, functions, and binding affinities for peptide ligands such as insulin and insulin-like growth factors (IGFs) (2–13). Structurally, each subunit in receptors is composed of three large protein fragments: the extracellular part (also known as the ectodomain), the intracellular part (containing kinase domains), and a single-pass transmembrane domain (TMD) that connects extracellular and intracellular fragments. Specifically, the TMD as well as the catalytic kinase domain are located in the *β*-chains of each subunit of receptor homodimers.

TMD potentially plays a critical role in mediating signaling *via* IR and IGF1R because ligand binding to extracellular subunits leads to conformational changes that are conveyed (*via* TMD) to kinase domains, thereby triggering trans-autophosphorylation and downstream signaling cascades (14–20). Initially, the TMD appeared to play a passive role in insulin signaling (21) but other studies indicate that modifications in TMDs of IR or IGF1R alter receptor internalization as well as affect kinase activation and negative cooperativity (22–25), while replacing IR–TMD with that of glycophorin A inhibits insulin action (26). The mechanistic details of these processes remain poorly understood at the molecular scale, but simple mechanical models for signal transduction *via* TMD suggest that a lateral shift or a rotational motion of TMD is energetically more favorable than the vertical motion in the phospholipid bilayer, as it would suggest dimerization of TMDs that could bring kinase domains in proximity (25, 27–29). However, recent studies propose different mechanisms for IR and IGF1R activation (3, 30): Lee et al. (31) have suggested that TMDs of IR in the non-activated basal state are constitutively dimerized and dissociate on ligand binding, while Kavran et al. (32) have suggested that ligand binding leads to dimerization of TMDs in IGF1R. Previously, a different “yo-yo” model of receptor activation was proposed by Ward et al. (10) in which the ligand-induced conformational change releases kinase domains (for transphosphorylation) from an initially constrained position near the membrane. These studies do not directly support a common mechanism of activation of transmembrane cell-surface receptors (27).

Therefore, the exact mechanism of signal transduction in IR and IGF1R remains elusive in part due to the lack of knowledge of intact structures of full-length receptors (in apo or ligand-bound forms) although several structures of excised extracellular and intracellular domains have been solved (33–48). The solution structure of IR–TMD has been determined in detergent micelles (49), but the deviation of the hydrophobic thickness of micelles from lipid bilayers can potentially cause changes in protein conformations (50). Nonetheless, this study suggested that the excised IR–TMD sequence remains largely monomeric in solution and forms an *α*-helix with a kink at residues Gly960 and Pro961, but the possibility of dimer formation was not excluded depending upon the detergent/protein ratio. It was also speculated that the presence of one SXXXG sequence motif in IR–TMD could play a role in dimerization similar to the GXXXG motif (51, 52). Currently, no experimental data on the structure of IGF1R–TMD are available.

We have previously shown that molecular dynamics (MD) simulations conducted in explicit solvent with all-atom structural models and enhanced sampling algorithms (53) are highly promising tools to understand conformational flexibility of receptor structures and their ligand-binding mechanisms (8, 54–58). In this work, we aim to study the structure, orientation, and conformational variability of TMDs of IR and IGF1R in an explicit lipid bilayer environment. In particular, we have studied the folding/unfolding behavior and stability of membrane-embedded peptide sequences of IR and IGF1R using enhanced sampling simulations conducted with metadynamics algorithm (59) because classical MD simulations are likely insufficient for sampling of all relevant peptide conformations in the lipid bilayer. In particular, our predicted structural ensembles are consistent with recent NMR data (49) and reveal that the presence of Gly960 and Pro961 in IR–TMD indeed results in increased flexibility in comparison to IGF1R–TMD, while metastable structural ensembles of both peptides show significant differences in their orientation in the membrane and in conformations of the N- and C-termini. We also observe different patterns of water distribution near peptide residues at the membrane–solvent interface and find that changes in backbone conformations of peptides correlate with certain angle variables measured relative to the membrane normal.

## 2 Materials and Methods

### 2.1 Molecular Dynamics Simulations: System Setup

**Figure 1:**
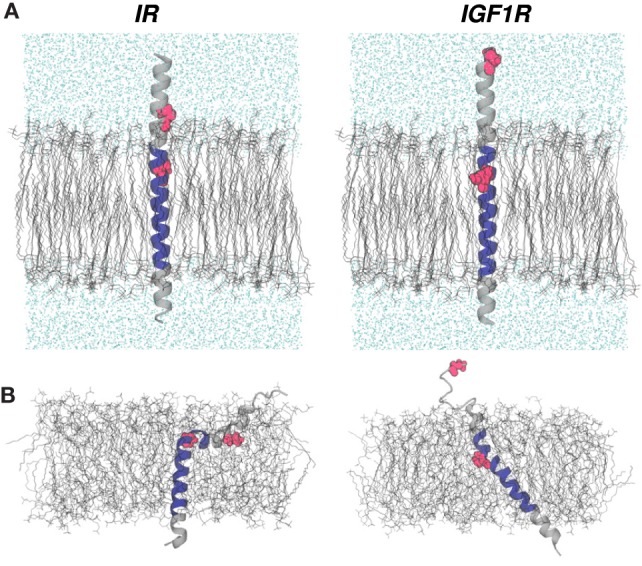
**(A)** Initial configurations of IR–TMD and IGF1R–TMD in membrane and solvent environments. Lipid and water molecules are shown in black and cyan wireframe representations, respectively. Peptides are shown as gray cartoons with the predicted TMD sequence rendered in blue. Two proline residues at the N-terminus of each peptide are shown in red space-filling representations. **(B)** Peptide configurations at the end of MD equilibration step 3 (see Materials and Methods).

All MD trajectories were generated with NAMD (60) using the TIP3P water model and the CHARMM force-field with the CMAP correction (61, 62). VMD was used for system creation, protein rendering, and analyses (63). All simulations were carried out in the NPT ensemble using the Langevin thermostat at 310 K and the Nosé-Hoover barostat. We modeled 51 residues for IR (939 – FYVTDYLDVPSNIAKIIIGPLIFVFLFSVVIGSIYLFL RKRQPDGPLG – 989) and IGF1R (918 – DPVFFYVQAKTGYENFIHLIIALPVAVLLIVGGLVIMLYVFHRKRNNSRLG – 968) that included the predicted TMD sequence (underlined; 957–979 for IR and 936–959 for IGF1R) for each receptor (Sequence numbering is based upon protein knowledgebase www.uniprot.org accession numbers P06213 and P08069). For each sequence, we generated an ideal *α*-helix as a starting structure using VMD’s psfgen tool and generated a palmitoyloleoylphosphatidylcholine (POPC) membrane patch of ~80 Å × 80 Å in size using VMD’s membrane builder tool. Each peptide was then embedded in the POPC bilayer by aligning the centers of mass and the principal axis of each helix along the *z*-direction. Thereafter, overlapping lipid molecules within 2 Å of each peptide were deleted. Each system was solvated with ~17700 water molecules, neutralized with KCl, and brought to an ionic strength of 0.2M. The final simulation domains measured ~83 Å × 80 Å × 140 Å and contained 74168 (IR) and 74144 (IGF1R) atoms, respectively. Each system was then equilibrated in three consecutive steps. In the first step, initially a conjugate-gradient minimization was carried out for 1000 cycles, which was followed by a short MD equilibration (0.5 ns long with a 2-fs time step) by keeping all atoms fixed except those in lipid tails. In the second step, MD equilibration was continued for 5 ns in the NPT ensemble by fixing only peptide atoms. In the third step, no atoms were fixed or constrained in a 50 ns MD equilibration in the NPT ensemble. The final atomic coordinates after the equilibration in the third step were used to setup enhanced exploration of peptide conformations in lipids using metadynamics, as described below. Initial and equilibrated configurations of IR–TMD and IGF1R–TMD are shown in Figure 1.

### 2.2 Metadynamics Simulations

Metadynamics is an enhanced sampling method for faster and uniform exploration of conformational space in a specified set of collective variables (CVs) by augmenting the force-field with a history-dependent biasing potential (*V*_meta_) of the following form (59, 64):

(1) $$V_{\text{meta}}(\xi \;)=\sum_{t^{\prime }=\tau_{\text{G}},2\tau_{\text{G}},\ldots }^{t^{\prime }<t}\;W\prod_{i=1}^{N_{\text{cv}}}\text{ exp}\left(-\frac{{\left(\xi_{i}-\xi_{i}(t^{\prime })\right)}^{2}}{2\delta {\;}_{\xi_{i}}^{2}}\right)$$

where *ξ_*i*_* is the current value of the CV, and *ξ_*i*_*(*t*′) is the value of the CV at time *t*′. *V*_meta_ is constructed as a sum of *N*_cv_-dimensional repulsive Gaussian functions with a chosen height (*W*) and width (*δ*). The Gaussian functions can be added at a desired frequency *τ*_G_. These three main parameters in metadynamics (*W*, *δ*, and *τ*_G_) control the efficiency and accuracy of the free energy reconstruction from converged metadynamics potential (*V*_meta_) (65). Metadynamics has been successfully applied to study many biophysical problems (66–72) including prediction of peptide conformations in lipid membranes (73, 74).

In this work, we have used as CV the root-mean-squared-deviation (RMSD) of the backbone C*_α_* atoms with respect to a perfect *α*-helix. The RMSD CV was bounded between 0 and 15 Å, and therefore, low values of RMSD indicate helical conformations and higher values indicate kinks and/or unfolded states. For all metadynamics simulations, a 1-fs integration time step was used, and the Gaussian height (*W*), width (*δ*), and frequency (*τ*_G_) of 0.1 kcal/mol, 0.2 Å, and 1 ps, respectively, were used. Metadynamics simulations converged in 160 (IR–TMD) and 145 ns (IGF1R–TMD), respectively, after which each trajectory sampled the CV range diffusively. The converged free-energy profiles from the last 10 ns of each metadynamics trajectory were used for analyzing metastable conformations and for carrying out other analyses reported in this work. We note that we have not studied the effect of including multiple CVs in our simulations. Additionally, we point out that the protonation states of all residues were assigned at physiological pH, and the effect of varying pH was not explicitly studied here.

## 3 Results

### 3.1 Free Energy Profiles and Conformational Ensembles of IR–TMD and IGF1R–TMD

Starting with a perfectly *α*-helical conformation of each peptide (Figure 1A), we carried out independent ~55 ns long MD equilibrations in explicit membrane and solvent environments before launching enhanced sampling simulations using metadynamics (see Materials and Methods). The final conformations of peptides sampled from these MD trajectories (Figure 1B) show that even at these short-timescales, peptides deviate from their initial conformations and adopt tilted conformational states with respect to the membrane normal. Specifically, IR–TMD largely maintains an *α*-helical structure but with a sharp kink at Pro961 such that residues 939–958 in the N-terminal helix interact strongly with lipids than the water molecules. IGF1R–TMD also remains *α*-helical with a minor kink at Pro941, but the first 10 residues in the N-terminus spontaneously unfold and interact with the water molecules. The C*_α_*-RMSDs relative to a perfect helix for the final peptide conformations are 6.35 Å (IR–TMD) and 3.75 Å (IGF1R–TMD), respectively.

**Figure 2:**
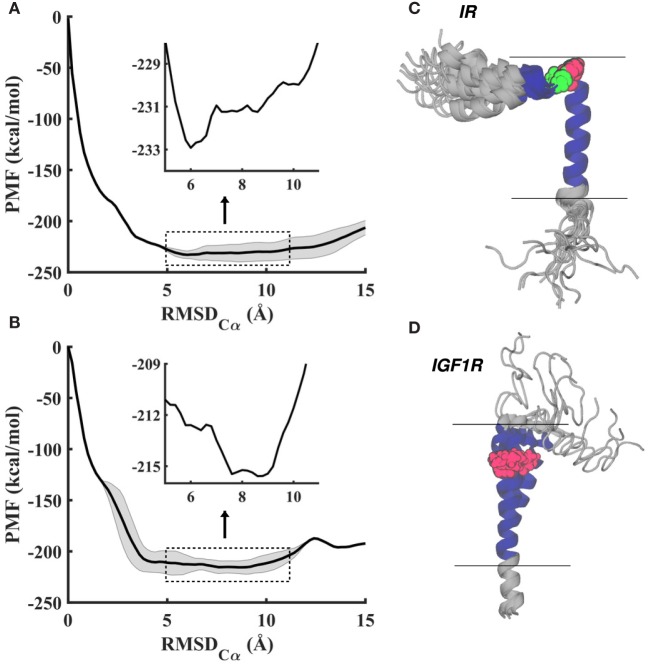
**(A,B)** Averaged potentials of mean force (PMFs) from the last 10 ns of metadynamics simulations for IR–TMD (top) and IGF1R–TMD (bottom) in a lipid membrane. Free energy profiles show relatively small energetic differences (~2–3 kcal/mol) in a wide range (5–11 Å) of RMSD, as indicated by magnified profiles (*inset*). Shaded regions on PMF traces indicate computed statistical variation in free energy profiles. **(C,D)** Overlay of all metastable/stable conformations for IR–TMD and IGF1R–TMD in gray cartoon representations except the transmembrane sequence, which is displayed in blue cartoons. All depicted conformations are aligned on the initial configuration of each peptide (shown in Figure 1A) with alignment based upon residues in the predicted transmembrane sequence (blue cartoon). Pro961 (IR–TMD) and Pro941 (IGF1R–TMD) are shown in red, and Gly960 (IR) in green space-filling representations, respectively. Horizontal lines indicate the approximate location of the lipid bilayer.

To uniformly explore peptide conformations between 0 and 15 Å RMSD and to obtain estimates on the free energy, we carried out 160 ns (IR–TMD) and 145 ns (IGF1R–TMD) long metadynamics simulations (see Materials and Methods). Consistent with enhanced conformational sampling, each peptide visited both helical and non-helical states multiple times during these simulations. The averaged free energy profiles (potentials of mean force; PMFs) from the last 10 ns of each metadynamics trajectory (Figures 2A,B) indicate that peptide conformations below an RMSD of ~3.5 Å and above ~11.5 Å are significantly higher in free energy relative to other states. This suggests that peptides prefer neither a fully helical structure (which occurs at 0 Å RMSD) nor a significantly unfolded configuration (which occurs beyond 12 Å RMSD), but instead metastable/stable configurations likely contain both helical and partially unfolded structural motifs. Moreover, the stable conformations with the lowest free-energy relative to other states occur at ~6 Å RMSD for IR–TMD and ~8 Å RMSD for IGF1R–TMD (*inset* in Figures 2A,B).

**Figure 3:**
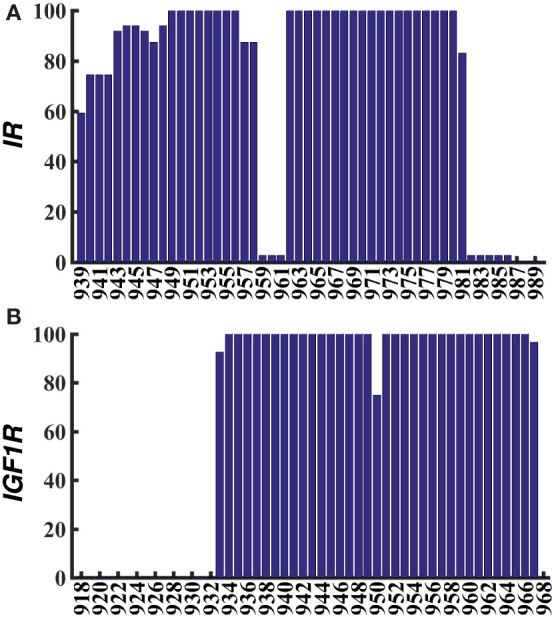
**Averaged percentage helicity per residue for all metastable/stable conformations of IR–TMD (A) and IGF1R–TMD (B)**.

From the last 10 ns of each metadynamics trajectory, we harvested several metastable/stable configurations for each peptide (17 for IR and 11 for IGF1R) with a ~2–3 kcal/mol free-energy difference. These conformations for IR–TMD and IGF1R–TMD are distinct (Figures 2C,D) and have the following features: (1) in IR–TMD, *α*-helical structures are observed for residues 939–958 in the N-terminus and residues 962–980 (part of the predicted transmembrane domain sequence, 957–980, of IR). These two helices are stably held together by a sharp kink at Gly960 and Pro961. The remaining residues in the C-terminus (981–989) are highly flexible and adopt unfolded conformations; and (2) in IGF1R–TMD, the N-terminal residues 918–932 are significantly flexible and unfolded, a small *α*-helix kinked at Pro941 is observed between 933 and 941, while a full *α*-helix is observed for residues 942–968. To quantify these observations, we further carried out secondary structure analysis on all metastable/stable configurations and computed average helicity on a per residue basis. These results (Figure 3) show that the *α*-helical content for IR–TMD is reduced between residues 939 and 942, and no helical content is observed between residues 959–961 and 982–989, while for IGF1R–TMD, no helical content is present between residues 918 and 931, and a minor decrease in helicity is observed at residue Gly950. We note that an unstable kink at Gly950 mostly switches back to a stable *α*-helix, as described in the following.

### 3.2 Orientation of IR–TMD and IGF1R–TMD in the Membrane

**Figure 4:**
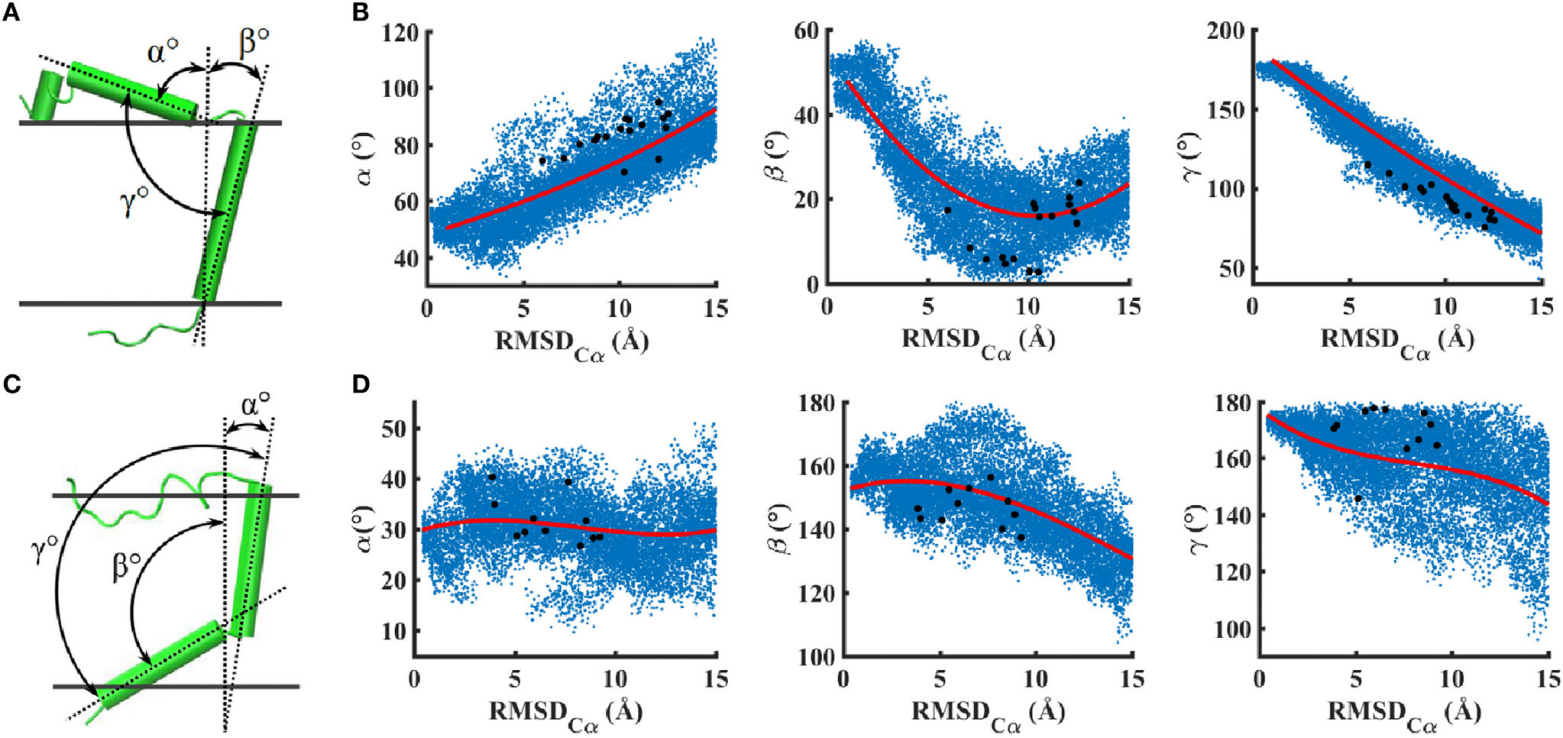
**Two angle collective variables relative to the membrane normal and one between the helices are shown to quantify peptide orientations in a lipid bilayer: (A,B) IR–TMD and (C,D) IGF1R–TMD**. Correlations of angles with RMSD are shown in **(B,D)**. Scattered blue dots indicate all values of angles explored *via* metadynamics trajectories, and red lines are the best fit curves. Black dots are angles corresponding to metastable/stables conformations shown in Figures 2C,D.

In metadynamics simulations, the change in RMSD of peptides relative to a perfect helix could be due to several different types of structural features such as tilting, bending, or unfolding. Therefore, to understand the orientation of peptides in the lipid bilayer, we computed three angle variables and analyzed their correlation with the RMSD change (Figure 4). For IR–TMD, *α* and *β* characterize the orientation (relative to the membrane normal) of the helix preceding Pro961 and the helix corresponding to the transmembrane sequence (962–979), and *γ* characterizes the interhelical angle, while in IGF1R–TMD, *α* and *β* characterize the orientation of helices between 934–948 and 951–966 relative to the membrane normal, and *γ* is the interhelical angle.

These data (Figures 4B,D) indicate that several conformations in the RMSD range (0–15 Å) can take a wide variety of angle values suggesting multiple orientations of peptides due to enhanced conformational sampling *via* metadynamics. For IR–TMD, we find that angles *α* and *γ* are correlated with RMSD such that an increase in RMSD results in an increase in *α* but a decrease in γ. Structurally, this means that the N-terminal helix in IR–TMD kinks toward the membrane, thereby becoming parallel to the membrane–solvent interface, while the membrane-embedded helix straightens to align along the membrane normal, as also indicated by a sharp decrease in *β*. For the metastable/stable conformations of IR (Figure 2C), we observe *α* values between ~70° and 90°, *γ* values slightly smaller than ~110°, and *β* values between ~5° and 25°. For IGF1R–TMD, we observe no significant correlation between the angle *α* and RMSD as *α* remains near 30° on average, suggesting that the helix between residues 934 and 948 remains tilted relative to the membrane normal. However, an increase in RMSD is correlated with a decrease in *β* and *γ* that leads to a kink at Gly950. This kink is unstable and not observed in metastable/stable conformations of IGF1R–TMD (Figure 2D) where *γ* values near 180° are observed. In these IGF1R–TMD conformations, a significant contribution to change in RMSD is due to the unfolding of the N-terminus (residues 918–932), and a minor contribution is due to a kink at Pro941.

### 3.3 Interactions of Peptides with the Solvent

**Figure 5:**
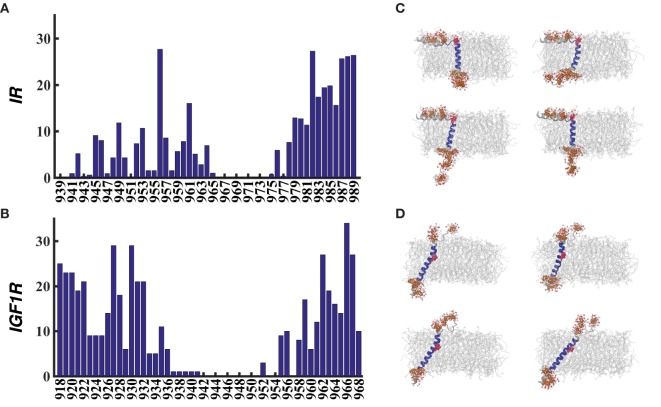
**(A,B)** The average number of water molecules per residue within ~4.5 Å of metastable/stable conformations of IR–TMD and IGF1R–TMD. **(C,D)** Selected snapshots from metastable/stable conformations of IR–TMD **(C)** and IGF1R–TMD **(D)** are shown to highlight interactions with water molecules (red licorice representations). Several charged residues in the termini of each peptide and a proline residue (961 for IR and 941 for IGF1R) are shown in brown and red space-filling representations, respectively. Each peptide is rendered as a cartoon in the same coloring scheme as in Figures 1 and 2.

In each 51-residue long peptide studied here, several charged amino acids are present in the sequence preceding as well as following the predicted TMD sequence (957–979 for IR and 936–959 for IGF1R). Because we observed kinked or unstructured configurations in the termini of each peptide, we analyzed all metastable conformations for interactions with water molecules at the membrane–solvent interface. Specifically, we present average number of water molecules within 4.5 Å of each protein residue in Figure 5. These data show that no water molecules are observed in the vicinity of helix-forming hydrophobic residues buried in the membrane (for example, 965–977 for IR–TMD and 937–954 for IGF1R–TMD). Both IR–TMD and IGF1R–TMD have an “Arg–Lys–Arg” motif immediately following the TMD sequence that is exposed to solvent as indicated by the increasing number of water molecules for residues in this motif of each peptide. Importantly, this motif is part of the unfolded C-terminus in IR–TMD but is fully folded in IGF1R–TMD. The exposure of this motif to solvent is compensated by a larger tilt angle in IGF1R–TMD in comparison to homologous sequence in IR–TMD. Several other residues in the C-terminus of each peptide have over 10 water molecules in their vicinity. A significant difference in water distribution is observed in the N-terminus of each peptide largely because residues 918–932 in IGF1R–TMD are highly flexible, unfolded, and located outside the membrane, while the homologous residues in IR–TMD form an *α*-helix resting at the membrane–solvent interface, such that a charged residue Lys956 has over 25 water molecules in its vicinity. The kink-forming residue Pro961 in IR–TMD is also significantly exposed to the solvent, but the corresponding residue Pro941 in IGF1R–TMD is completely shielded from the solvent. The highest water density is observed for Arg966 in IGF1R–TMD, and for Lys956 or Arg982 in IR–TMD.

## 4 Discussion

In this work, we have presented all-atom structural models of 51-residue long peptides containing the transmembrane domain sequence of IR and IGF1R (957–979 for IR and 936–959 for IGF1R; see Materials and Methods). These models have been generated in explicit membrane and solvent environments using MD simulations assisted by enhanced conformational sampling algorithms that facilitate extensive sampling of conformational space and provide information on key thermodynamic properties such as the free energy. For both receptors, we observe that the residues corresponding to the transmembrane domain sequence are fully membrane-embedded and form *α*-helices with a major kink at Pro961 in IR and a minor kink at Pro941 in IGF1R. A kink in IGF1R–TMD at Gly950 is unstable and recovers to an *α*-helical conformation. Based upon angle collective variables characterizing the orientation of each peptide in the membrane (Figure 4), we observe that the membrane-embedded *α*-helix in IGF1R–TMD is significantly more tilted (relative to the membrane normal) than in IR–TMD (Figures 5C,D). However, it is important to point out that these angles were not explicitly included as CVs in our metadynamics calculations and therefore were not extensively sampled. The values of angles reported in Figure 4 are those that correspond to extensive sampling along RMSD CV, as also indicated by multiple values of a specific angle corresponding to a single value of RMSD.

We also notice major differences in conformations of peptide termini: a short *α*-helix is observed for the N-terminal residues (939–958) of IR–TMD, but significantly unfolded and flexible conformations are observed for the N-terminal residues (918–932) of IGF1R–TMD, while an *α*-helix is observed for the C-terminal residues (960–968) of IGF1R, but unfolded and flexible conformations are observed for the C-terminal residues (981–989) of IR. Importantly, irrespective of different conformations in the C-terminus of each peptide, an “Arg–Lys–Arg” motif is solvent exposed, albeit at the expense of a larger tilt angle in IGF1R–TMD than in IR–TMD. However, we observe that all N-terminal residues (918–932) in IGF1R–TMD are solvent exposed, but only a few N-terminal residues (Lys956 and Pro961) in IR–TMD are significantly solvated. This difference is primarily due to the fact that a short *α*-helix in the N-terminus of IR–TMD is partially membrane-embedded such that the positively charged residues are oriented toward the membrane–solvent interface, while the N-terminal residues in many metastable conformations of IGF1R–TMD reside outside the membrane.

Li et al. (49) have recently determined a solution structure of the transmembrane domain of IR (PDB code 2MFR) using NMR spectroscopy in dodecylphosphocholine (DPC) micelles. The following features observed in the NMR conformational ensemble are consistent with our IR–TMD structural models: (i) a well-defined *α*-helix (between residues Leu962 and Tyr976) buried in the DPC micelles with a kink at Gly960 and Pro961; (ii) a flexible and solvent-exposed C-terminal region (between residues Gln983 and Leu988); and (iii) a short *α*-helix (between residues Phe939 and Tyr944) partially buried in the DPC micelles with weak solvent interactions for Thr942 and Asp943. On comparing our models with the NMR structure, we observe that the kink angle at Pro961 in our IR–TMD models is larger than what is observed in the NMR structure, which results in increased interactions of Pro961 with the solvent in our models. We therefore analyzed the spherical micellar region encasing IR–TMD reported in Li et al.’s work (49) and found that it is at least ~3–4 Å thicker than the equilibrium thickness of a POPC membrane. We speculate that the difference in the hydrophobic thickness of a bilayer and a micelle could have contributed to a difference in the kink-angle near Gly960 and Pro961. However, the observation of a kink at these residues in our models and the NMR structure is consistent with the observation of enhanced helicity in IR–TMD on individual or simultaneous mutations of Gly960 and Pro961 to Ala (75) as well as with the role of Gly and Pro residues as helix breakers (76, 77). Currently, no experimental structure of IGF1R–TMD is known, but consistent with our all-atom structural models of IGF1R–TMD, classical MD simulations reported in Kavran et al.’s work (32) indicate a kink at Pro941, interactions of His935 with the solvent, and a significantly tilted *α*-helical conformation of the transmembrane sequence.

A major unresolved question is related to the dimerization of IR–TMD and IGF1R–TMD in the basal or activated states of receptors in part because no experimental structures of these domains in a dimeric configuration have been reported so far. However, different models have been proposed (3, 30) by Ward et al. (10), Lee et al. (31), and Kavran et al. (32), as outlined in the introduction. For the isolated IR–TMD, Li et al. (49) primarily observed a monomeric conformation in the DPC micelles but suggested the possibility of a dimer with weak binding affinity because replacing IR–TMD with a strong dimer-forming TMD of glycophorin A (78) inhibits insulin signaling (26). For IGF1R–TMD, Kavran et al.’s work (32) has suggested that IGF1R–TMD can form stable dimers by associating near kink-inducing residue Pro941 such that His935 residues in helices can interact with each other and the solvent. The conformations of helices reported in this dimer are consistent with our IGF1R–TMD structural models. Importantly, Cabail et al. (33) have provided crystallographic, biochemical, and biophysical evidence showing that the phosphorylated kinase domains of IR and IGF1R dimerize through exchanged juxtamembrane regions, but ~20 residues of unknown structure in the N-terminus of the juxtamembrane sequence preclude conclusive support for dimerized or dissociated transmembrane helices. While we have not directly studied the dimerization of IR–TMD or IGF1R–TMD in this work, the C-terminal sequences in our 51-residue long peptides include several residues from the N-terminal juxtamembrane regions of receptors (10 residues of IR and 9 residues for IGF1R). As described above, in both receptors, these residues are significantly exposed to the solvent, but adopt distinct conformations (in IR–TMD, completely unstructured and flexible conformations are observed as opposed to IGF1R–TMD, where these residues participate in an *α*-helix). We speculate that these conformational differences could contribute to different structural organization of kinase domains in the basal or activated states of receptors.

## 5 Conclusion

Using MD simulations combined with enhanced sampling algorithms, we have presented all-atom structural models of IR–TMD and IGF1R–TMD in explicit membrane and solvent environments. We found intact *α*-helical conformations for the membrane-embedded residues of each peptide with a larger tilt-angle (relative to the membrane normal) in IGF1R–TMD in comparison to IR–TMD. We also observe kinks in membrane-spanning helices at Pro961 (IR) and Pro941 (IGF1R). The major differences in peptide conformations are in the terminal sequences where a kinked *α*-helix is observed for the N-terminus of IR–TMD as opposed to unfolded conformations in IGF1R, and an *α*-helix is observed in the C-terminus of IGF1R–TMD as opposed to unfolded conformations in IR–TMD. These differences in conformations lead to increased solvation of the N-terminal residues in IGF1R–TMD in comparison to IR–TMD, but similar solvation patterns are observed in the C-terminal residues containing an “Arg–Lys–Arg” motif.

## Author Contributions

HM and HV conceived and designed research; HM performed research and analyzed data; HM and HV wrote the paper.

## Conflict of Interest Statement

The authors declare that the research was conducted in the absence of any commercial or financial relationships that could be construed as a potential conflict of interest.
